# Positive association of collagen type I with non-muscle invasive bladder cancer progression

**DOI:** 10.18632/oncotarget.12089

**Published:** 2016-09-17

**Authors:** Michael Brooks, Qianxing Mo, Ross Krasnow, Philip Levy Ho, Yu-Cheng Lee, Jing Xiao, Antonina Kurtova, Seth Lerner, Gui Godoy, Weiguo Jian, Patricia Castro, Fengju Chen, David Rowley, Michael Ittmann, Keith Syson Chan

**Affiliations:** ^1^ Scott Department of Urology, Baylor College of Medicine, One Baylor Plaza, Houston, Texas, 77030; ^2^ Department of Medicine, Baylor College of Medicine, One Baylor Plaza, Houston, Texas, 77030; ^3^ Department of Urology, Baylor College of Medicine, Kelsey-Seybold Clinic, Houston, Texas 77030; ^4^ Department of Molecular & Cellular Biology, Baylor College of Medicine, One Baylor Plaza, Houston, Texas, 77030; ^5^ Department of Pathology and Immunology, and Michael E. DeBakey VAMC, Baylor College of Medicine, One Baylor Plaza, Houston, Texas, 77030; ^6^ Dan L Duncan Cancer Center, Baylor College of Medicine, One Baylor Plaza, Houston, Texas, 77030; ^7^ Center for Cell, Gene and Therapy, Baylor College of Medicine, One Baylor Plaza, Houston, Texas, 77030; ^8^ Center for Drug Discovery Baylor College of Medicine, One Baylor Plaza, Houston, Texas, 77030

**Keywords:** bladder cancer, type I collagen, tumor microenvironment, invasion, cancer progression

## Abstract

**PURPOSE:**

Non-muscle invasive bladder cancers (NMIBC) are generally curable, while ~15% progresses into muscle-invasive cancer with poor prognosis. While efforts have been made to identify genetic alternations associated with progression, the extracellular matrix (ECM) microenvironment remains largely unexplored. Type I collagen is a major component of the bladder ECM, and can be altered during cancer progression. We set out to explore the association of type I collagen with NMIBC progression.

**EXPERIMENTAL DESIGN:**

The associations of *COL1A1* and *COL1A2* mRNA levels with progression were evaluated in a multi-center cohort of 189 patients with NMIBCs. Type I collagen protein expression and structure were evaluated in an independent single-center cohort of 80 patients with NMIBCs. Immunohistochemical analysis was performed and state-of-the-art multi-photon microscopy was used to evaluate collagen structure via second harmonic generation imaging. Progression to muscle invasion was the primary outcome. Kaplan-Meier method, Cox regression, and Wilcoxon rank-sum were used for statistical analysis.

**RESULTS:**

There is a significant association of high *COL1A1* and *COL1A2* mRNA expression in patients with poor progression-free survival (*P*=0.0037 and *P*=0.011, respectively) and overall survival (*P*=0.024 and *P*=0.012, respectively). Additionally, immunohistochemistry analysis of type I collagen protein deposition revealed a significant association with progression (*P*=0.0145); Second-harmonic generation imaging revealed a significant lower collagen fiber curvature ratio in patients with invasive progression (*P* = 0.0018).

**CONCLUSIONS:**

Alterations in the ECM microenvironment, particularly type I collagen, likely contributes to bladder cancer progression. These findings will open avenues to future functional studies to investigate ECM-tumor interaction as a potential therapeutic intervention to treat NMIBCs.

## INTRODUCTION

Approximately 400,000 new bladder cancer cases are diagnosed each year worldwide, with the highest rates in Europe, United States and Egypt [1]. While non- muscle invasive bladder cancer (NMIBC) recurs in up to 70% of cases after local treatment, they generally do not cause mortality [2]. However, once progression occurs (i.e. invasion into smooth muscle layers), rates of cancer-specific survival decrease to approximately 65% even in organ-confined disease [3, 4]. For this reason, most recent large-scale clinical trials define progression as increase in tumor staging from NMIBC to ≥T2 muscle-invasive bladder cancer (MIBC) [5–7]. Despite extensive research efforts to define genetic alterations and molecular signatures that associated with progression [8–11], the underlying mechanisms of bladder cancer progression remain elusive.

Several alterations within urothelial carcinoma cells were reported to associate with NMIBC progression. For instance, FGFR3 activating mutation associates with and is predictive of better prognosis [12–14], and abnormal expression of cytokeratin 20 positively associates with progression [15, 16]. Methylation of TBX2 and TBX3 genes are recently demonstrated to be predictive of progression [17]. Nevertheless, the tumor microenvironment, specifically alterations in the subepithelial stroma, during invasive progression of NMIBC remains largely unexplored.

It is well appreciated that epithelial cell functions, including cellular differentiation, migration and invasion, are directed by physical interactions with the extracellular matrix (ECM) [18]. Type I collagen is a major structural component of the ECM within bladder, and epithelial tumorigenesis is often accompanied by ECM alterations and remodeling [19]. In the current study, we set out to investigate a possible association of ECM alteration, particularly type I collagen mRNA and protein expression, with NMIBC progression to muscle-invasive disease.

## RESULTS

### High *COL1A1* and *COL1A2* mRNA expression correlates with poor overall and progression-free survival

**Figure 1:**
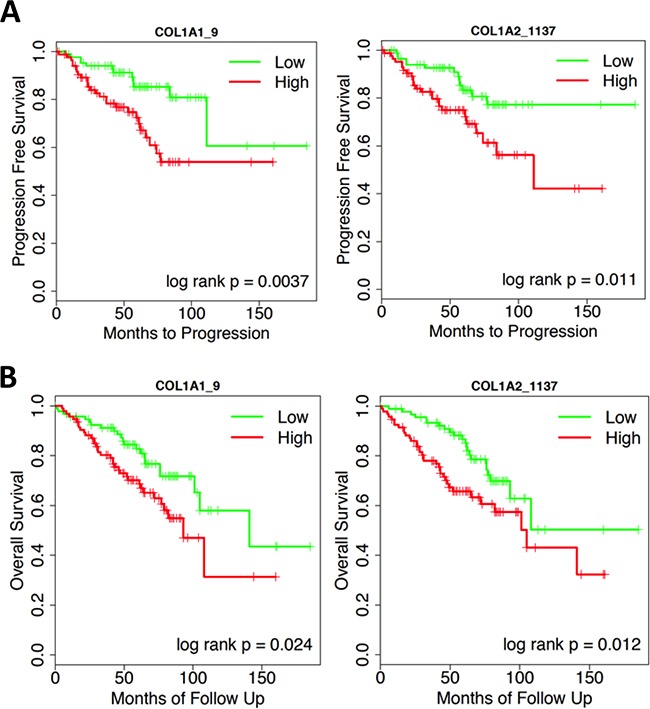
Representative Kaplan-Meier analyses of the A. progression-free and, B. overall survival corresponding to low *(green) or high (red) mRNA expression of COL1A1 and COL1A2*

First, we analyzed gene expression of a multi-institutional cohort comprising 189 Ta NMIBC patients, mRNA expression of genes encoding collagen I were evaluated in correlative with patient progression and survival using Cox regression and Kaplan-Meier analyses. Increased Type I collagen mRNA expression (*COL1A1* and *COL1A2* genes) was associated with NMIBC progression in this multi-institutional cohort (Figure 1A; patient information summarized in Table 1). Cox regression analyses demonstrated that higher *COL1A1* and *COL1A2* mRNA expression in patients were significantly associated with worse progression-free survival (Figure 1A) and overall survival (Figure 1B). Ten out of the fourteen *COL1A1* probes and four out of the six *COL1A2* probes demonstrated statistical significance for progression-free survival (p < 0.05 and p < 0.01, respectively - Supplementary Table S1). For overall survival, 9 out of the 14 *COL1A1* probes and 4 out of the 6 *COL1A2* probes demonstrated statistical significance (p < 0.01 and p < 0.001, respectively - Supplementary Table S1). Representative Kaplan-Meier analyses of the progression-free and overall survival corresponding to low or high mRNA expression of *COL1A1* and *COL1A2* are illustrated in Figure 1A and Figure 1B, respectively.

**Table 1:** Stage Tis, Ta mRNA Cohort – 189 Patients

Characteristic	N (%)
Median Age (IQR)	67 (58-74)
Male	154 (81)
High Grade*	64 (34)
CIS+	17 (9)
EORTC High-Risk Group	67 (35)
Median Follow-up in Years (IQR)	4.8 (3.4-6.7)
Progression to T2	21 (11)
Deaths	57 (30)

* 2004 World Health Organization grade definitions

** European Organisation for Research and Treatment of Cancer

### High collagen I protein expression near the tumor-ECM boundary significantly correlates with poor progression-free survival

**Figure 2:**
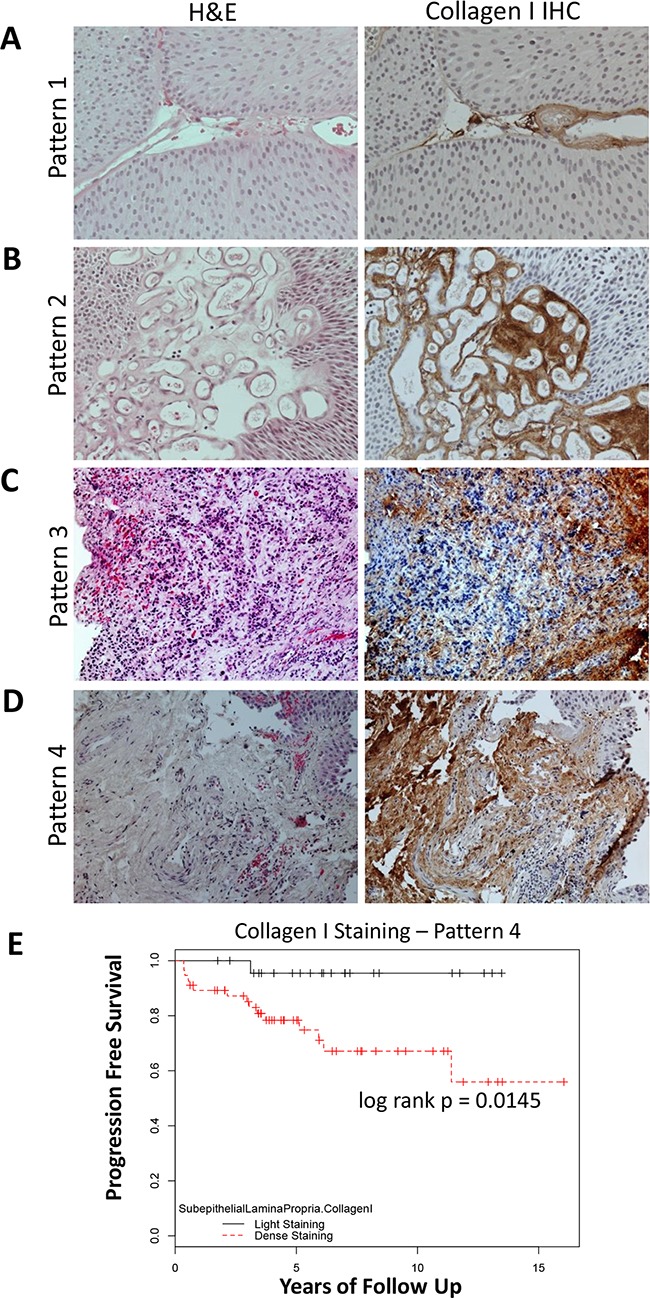
Specimen were scored based upon observed patterns of collagen I staining Representative images for each of the staining pattern were listed as: **A.** pattern type 1 – staining of thin papillary tumor stroma, **B.** pattern type 2 – staining of vascular tumor stroma, **C.** pattern type 3 – reticular staining pattern of collagen surrounding epithelial tumor cells, and **D.** pattern type 4 – dense staining of subepithelial lamina propria near the tumor-ECM boundary. Serial sections with Hematoxylin & Eosin (H&E) staining was demonstrated in the left column. **E.** Kaplan-Meier plot of Collagen I IHC protein expression in the LP and its association with progression-free survival, (*P* = 0.0145).

To evaluate type I collagen expression at the protein level, we performed immunohistochemical (IHC) analysis using a NMIBC patient cohort treated at a single institute (patient information summarized in Table 2). Immunohistochemical analysis revealed very specific brown 3,3′-Diaminobenzidine (DAB) staining of collagen I, evidently accompanied by negatively stained areas that were counterstained by Hematoxylin (blue; Figure 2A-2D). Four different staining patterns of type I collagen protein were observed (Figure 2A-2D). For staining pattern 1, collagen I was expressed within the thin lining of stromal regions within epithelial papillary lesions (Figure 2A); for staining pattern 2, collagen I was expressed within the vasculature of stroma (Figure 2B); for staining pattern 3, reticular expression of collagen I was observed surrounding epithelial tumor cells (Figure 2C); and, for staining pattern 4, dense collagen I was expressed within subepithelial lamina propria near the tumor-ECM boundary (Figure 2D). Particularly, increased collagen I protein expression near the tumor-ECM boundary (staining pattern 4), was significantly associated with progression of NMIBC in this single institution cohort (Figure 2E; p = 0.0145). Representative patient samples with comparison to serial sections of H&E staining are also demonstrated in Figure 2A-2D. Nevertheless, collagen I staining within papillary tumor stroma, vascular tumor stroma, or surrounding reticular stroma (staining pattern types 1-3, respectively) were not significantly associated with NMIBC progression.

**Table 2:** Stage Tis, Ta Risk-Matched Tumor Bank Cohort – 80 Patients

Characteristic	Progression (n = 16) N (%)	No Progression (n = 64) N (%)	P-Value
Median Age (IQR)	74 (60-78)	69 (62-78)	0.66
Male	16 (100)	63 (98)	1.00
High Grade*	9 (56)	37 (58)	0.91
CIS+	9 (56)	27 (42)	0.31
Multifocal	10 (63)	19 (30)	0.01
> 3 cm	3 (19)	10 (16)	0.76
EORTC High-Risk Group**	9 (56)	37 (58)	0.91
Prior Local Treatment	1 (6)	5 (8)	1.00
Median Follow-up in Years (IQR)	6.6 (4.6-10.7)	5.5 (3.5-8.4)	0.32

* 2004 World Health Organization grade definitions

** European Organisation for Research and Treatment of Cancer

### Second harmonic generation (SHG) imaging revealed a significant association of low collagen fiber curvature ratio to progression

**Figure 3:**
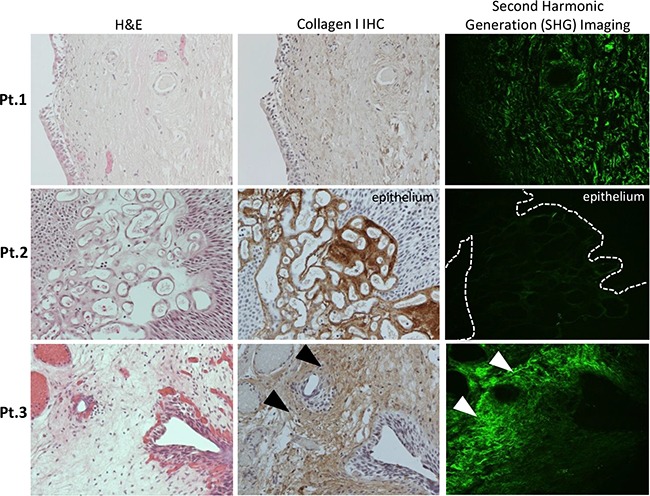
Representative images from individual NMIBC patients demonstrating Hematoxylin & Eosin (H&E) analysis, Collagen I protein expression by immunohistochemical analysis (IHC) and Second Harmonic Generation (SHG) imaging (20x serial sections from left to right) Patient 1 (Pt.1) – Carcinoma-*in-situ* (CIS) with normal lamina propria (LP), collagen I staining by IHC and SHG imaging of curved collagen fibers, no progression. Patient 2 (Pt.2) – Papillary tumor with increased collagen I IHC staining within vascular stroma, also visualized on SHG, no progression. Patient 3 (Pt.3)– increased sub-epithelial LP staining and fibers with low curvature ratio by SHG imaging, experienced progression to MIBC. Corresponding areas of dense, straight-fibered collagen I deposition in the subepithelial stroma were marked with arrows.

**Figure 4:**
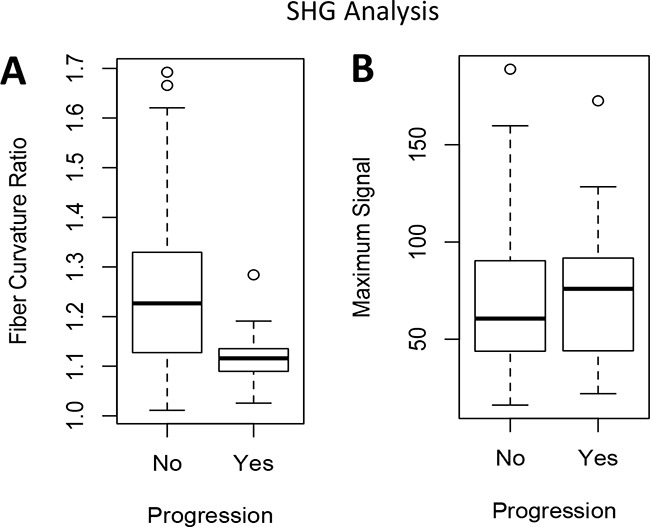
SHG imaging quantification comparing patients with progression to muscle-invasive disease versus those with no progression Wilcoxon rank sum was used to compare measurements between patients with and without progression. **(A)** Median fiber curvature ratio *P* = 0.0018 **(B)** Maximum SHG signal P = 0.652.

Due to the physical properties of type I collagen, when excited with a 400 nm wavelength photon, type I collagen emits a photon at exactly twice the wavelength (800 nm) [20]. This unique endogenous property of collagen can be visualized using state-of-the-art two-photon confocal microscopy, with a technique called SHG imaging [20]. Therefore, this imaging modality can reveal the structure of non-denatured collagen type I in tumor tissues without the aid of staining or fluorescent probes. The structure of subepithelial type I collagen was further examined using SHG imaging, using serial sections from the prior specimens concurrently analyzed by IHC (Figure 3; H&E and ColI IHC; representative images from patient 1-3). We have quantified collagen I structure in these SHG images (Figure 3; green fluorescent pseudo color), using a previously reported methodology to measure the curvature ratio (CR) of individual collagen fibers [21]. The CR was calculated using a previously reported method, where the traced length of the fibril (A) is divided by the linear distance between the two ends (B) [21, 22]. The median fibril CR (A divided by B) was compared between each image (Figure 3). Significantly lower collagen fiber CR near the tumor-stromal interface was observed in NMIBC specimen that progress into MIBC, when compared to those that did not progress into MIBC (Figure 4A; p = 0.0018). However, there was no significant difference in the maximum SHG signal between these two patient subgroups (Figure 4B).

## DISCUSSION

Recently, it has been reported that type I collagen remodeling in the ECM microenvironment accompanies stromal invasion and epithelial tumorigenesis in other cancer types [22]. For the first time, we have investigated the collagen microenvironment in the context of NMIBC progression, and demonstrated a positive association of high *COL1A1* and *COL1A2* mRNA expression to progression and decreased overall survival in non-muscle invasive (Ta and Tis) disease (Figure 1) in a multi-center cohort. Within this multi-center NMIBC cohort, 35% of patients were considered high-risk based upon The European Organization for Research and Treatment of Cancer (EORTC) classification (Table 1), and risk group was significantly associated with progression and overall survival, as expected. Therefore, we decided to investigate the association of altered type I collagen protein expression with NMIBC progression in another risk-matched cohort of NMIBC. FFPE tissue blocks from the Michael E. DeBakey VAMC tissue bank, from 1992-2012, were obtained for this purpose. The control group was well-matched to the progression group, as the comparison between the risk factors revealed no major differences (Table 2), while multifocal disease was the only factor statistically increased in the progression group (Table 2). The VAMC treatment setting was advantageous for this investigation for several reasons. First, with the exception of six patients who underwent resection at an outside facility prior to their initial presentation to the VAMC, all tissues were obtained from the initial diagnosis and local surgical resection. Therefore, stromal changes associated with prior local surgical or intravesical treatment-induced wound healing would not be a confounding factor in this cohort. Second, the majority of patients in the cohort were followed long-term at the VAMC, allowing for detailed clinical follow-up.

Using these high-quality banked FFPE samples, type I collagen IHC was performed on serially sectioned slides, using IHC and a novel imaging analysis. Two blinded observers applied previously determined scoring criteria (Figure 2A-2D). To our knowledge at the time of this study, no other study has described patterns of bladder tumor type I collagen staining. Therefore, the criteria for papillary tumor stromal staining, vascular tumor stromal staining, reticular tumor stromal staining, and dense lamina propria staining near the tumor-ECM boundary (staining patterns 1-4, respectively, Figure 2A-2D), were based on our objective observation. In our analysis, dense type I collagen staining in the subepithelial lamina propria near the tumor-ECM boundary, was significantly associated with progression (Figure 2E). Similar collagen I staining patterns was observed in colon cancer at the invasive fronts [23]. In the context of bladder cancer, our IHC findings were logical for a few reasons. First, papillary tumors with fine stromal projections were documented to be less prone to progression when compared to broad-based or sessile tumors [24]. Secondly, the lamina propria is the immediate adjacent site at the invasive fronts for cancer cells; therefore, it is possible that type I collagen deposition within the lamina propria precedes muscle invasion.

We further investigated type I collagen structure in the context of these IHC staining pattern using SHG imaging. By comparing NMIBC tissues stained by collagen type I IHC to serially sectioned tumor tissues analyzed by SHG imaging (Figure 3; second and third column, respectively), we demonstrated the feasibility in using SHG imaging to capture collagen expressing areas within the bladder cancer specimens (Figure 3; third column). Additionally, SHG imaging provided unique quantitative data on the collagen type I fibril structure, where low CR (straighter fibers) were associated with tumor progression. Straighter fibers, with low CR, have been reported to represent a stronger tensile strength due to collagen fiber cross-linking and are associated with epithelial cancer cell invasion [22]. In prior research using breast cancer specimen derived from mouse models, these dense aligned type I collagen fibers at the tumor-ECM boundary led to increased rates of cancer progression [25–27]. Additional clinical research in breast cancer has demonstrated this type of pattern may provide valuable prognostic information in predicting clinical outcomes [28]. Consistent with this finding, we found a similar association in NMIBC, where patients with specimen demonstrating low CR experienced stage progression during follow-up.

Since type I collagen is one of the most abundant ECM components, these findings implicate a role for the stromal microenvironment in modulating invasive progression of NMIBC. Such implication is further supported by other study demonstrating additional collagen types, e.g. Col4a1 and Col18a1, are within a 12-gene signature predictive of progression [29]. Additionally, recent reports demonstrated a reciprocal interaction between urothelial carcinoma cells and the stromal compartment, through paracrine signaling pathways sonic-hedgehog and Wnt/BMP, which modulates epithelial differentiation and progression of bladder cancer [30, 31]. Indeed, not only have similar signaling pathways been described in other epithelial cancer types, but they can be modulated via epithelial transmembrane collagen receptors interacting with type I collagen in the ECM, resulting in epithelial mesenchymal transition (EMT) and progression of disease [23, 32, 33].

With respect to cellular differentiation, type I collagen can inhibit tumor differentiation in colon cancer models, resulting in stem-cell like gene expression at the invasive front [32, 34]. Although these mechanisms have yet to be explored in bladder cancer, stem-cell like features are typically associated with more aggressive bladder cancer phenotypes, resulting in poor clinical outcomes [35, 36]. A very recent study performing high-throughput analysis of NMIBC revealed a Class 2 tumor that associates with progression, these Class 2 tumors enriched for cancer stem cell markers such as ALDH1A1, ALDH1A2, PROM1, NES and THY1. [37] Additionally, our group recently demonstrated that deposition of collagen-rich ECM, as part of the wound repair process, is exploited by bladder cancer cells contributing to progressive development of chemoresistance in invasive bladder cancer through a stem-cell like phenotype [38]. Therefore, further characterization of type I collagen in the ECM of bladder tumors, and the signaling pathways utilized for stromal-epithelial interaction, will provide insights into both progression of bladder cancer, and potential resistance to treatment.

Notwithstanding, we recognize that the current study could be limited by a retrospective design and cohort size. We used rigorous statistical methods, however due to our limited cohort size, we relied on univariate analysis to assess the association of type I collagen with NMIBC progression and overall survival. Although we attempted to correct for confounders in our risk-matched cohort, it would be of importance to investigate these findings in the future using a larger, prospective cohort via multivariate analysis.

Together, we demonstrated for the first time, alterations in type I collagen mRNA expression, protein expression, and collagen structure were associated with progression of NMIBC to muscle-invasion. Our findings implicate the importance of stroma and wound response during bladder tumorigenesis, and will likely open a new avenue to further study collagen signaling through integrins or other downstream collagen receptors/effectors during bladder tumorigenesis. Additionally, further evaluation of the tumor microenvironment and tumor stroma may reveal additional biomarkers for progression that could be used to aid existing nomograms for NMIBC risk prediction [39, 40].

## MATERIALS AND METHODS

### Patients

A multi-center NMIBC cohort comprising 189 Ta NMIBC patients was utilized to investigate the association between collagen type I mRNA expression levels (*COL1A1* and *COL1A2*) and clinical outcome. The patient characteristics for this cohort are described in Table 1. These patients were part of a multi-institutional effort to validate an mRNA assay predicting clinical outcomes [41]. Tumor progression was defined as increase in stage to T2 (muscle-invasive disease) or higher on subsequent biopsy or development of clinically proven metastasis. Since pathological tumor progression was our primary study endpoint, patients with initial stage T1 disease (lamina propria invasion) or higher were excluded due to the approximate 15-30% incidence of initial under staging of T1 disease [42–44].

A second single-institution NMIBC cohort was developed to investigate type I collagen protein expression and structure. From 1992 to 2012, 389 patients with bladder cancer who underwent local surgical treatment for bladder cancer during their initial presentation at the Michael E. DeBakey Veterans Affairs Medical Center (VAMC) in Houston were prospectively entered into an institutional cancer registry. Tumor stage and grade, as well as patient demographic information were entered into this registry. During this time period, sixteen patients who initially presented with Ta or Tis disease, experienced progression into muscle invasive disease during follow up. For each patient whom experienced progression, four other patients whom also presented with Ta or Tis disease during the same time period were included in this cohort. These patients had similar follow-up periods and known disease risk factors for progression including grade, presence of carcinoma *in situ*, tumors larger than 3 cm, and multifocal disease [2]. The patient characteristics for this cohort are described in Table 2.

### Specimen characteristics

For the single-institution cohort, tissues from their initial and subsequent surgical treatments were stored as formalin-fixed paraffin-embedded (FFPE) samples in a central tumor bank. After reviewing patient demographic information and clinical follow-up to ensure fidelity, FFPE tissues from the initial surgical resection (trans-urethral resection of the tumor) were obtained from a central tissue repository. All initial pathology reports were reviewed, and specimen were re-evaluated using haematoxylin and eosin (H&E) slides by a pathologist (M.I.) to confirm the accuracy of initial disease staging. The tissue repository and patient information were protected and stored in accordance with the good research practices and under approval of the institutional review board protocol number H-26809.

### Assay methods

mRNA expression from the multi-institutional cohort was analyzed using a custom cDNA microarray [41], with 14 and 6 specific probes used to hybridize *COL1A1* and *COL1A2* respectively (Figure 1).

For the single-institution cohort at the Michael E. DeBakey Veterans Affairs Medical Center (VAMC), immunohistochemistical (IHC) analysis was performed using a rabbit polyclonal antibody against Collagen I (Abcam, ab34710, 1:200). DAKO HRP-linked anti-rabbit secondary antibody was used to visualize the primary antibody. Collagen I IHC staining of tumor papillary stroma and subepithelial lamina propria compartments were compared, by two blinded observers. Specimen were scored based upon observed patterns of collagen I staining as follows: pattern type 1– staining of thin papillary tumor stroma, pattern type 2 – staining of vascular tumor stroma, pattern type 3 – reticular staining pattern of collagen surrounding epithelial tumor cells, and pattern type 4 – dense staining of subepithelial lamina propria near the tumor-ECM boundary (Figure 2A-2D). Both primary and secondary staining patterns were recorded for each specimen. Any discrepancies between observers were discussed until a final consensus score was agreed upon.

For second harmonic generation (SHG) imaging analysis, serial sections from the paraffin-embedded specimen were imaged using a state-of the art Carl Zeiss LSM 7 multiphoton microscope, and Chameleon Laser. The excitation wavelength was set to 400 nm, and imaging wavelength at 800 nm. Each specimen was imaged in at least two representative areas of stroma using a 20x objective. Images were analyzed using ImageJ version 1.48 (Wayne Rasband, NIH). Uniform 100x100 pixel regions of interest were repeatedly measured until the maximum signal was found for each image. The curvature ratio (CR) of individual collagen fibrils was measured for at least 10 representative fibrils in each image. The CR was calculated using a previously reported method, where the traced length of the fibril (A) is divided by the linear distance between the two ends (B) [21, 22]. The median fibril CR (A divided by B) was compared between each image (Figure 3).

### Study design

Follow-up data and patient characteristics for the multi-institutional cohort were collected in a retrospective fashion [41]. Therefore, the association of *COL1A1* and *COL1A2* mRNA expression with tumor progression and overall survival was examined using subgroup analysis of a retrospective cohort study.

For the single-institution cohort, although specimens and patient characteristics were prospectively entered into a tumor registry, follow-up information was obtained in a retrospective fashion. Thus, the investigation of collagen type I protein expression and structure was performed as a separate retrospective cohort study. As stated previously, tumor grade and staging, patient characteristics, and follow-up were matched between sixteen patients whom experienced progression and sixty-four patients who did not.

The primary endpoint for analysis was progression to muscle-invasive disease (stage T2 or higher), which was determined by subsequent tumor biopsy in the majority of cases, or in a few, development of metastasis. The secondary endpoint for analysis was overall survival. The median follow-up for the multi-institutional cohort was 4.1 years. Within the single-institution cohort, a median follow up of 6.6 years and 5.5 years, were observed for patients whom experienced progression and those who did not, respectively.

### Statistical analysis methods

All statistics were performed using R version 3.1.3 (https://cran.r-project.org/). Cox proportional hazard regression analysis was first used to examine if gene expression was significantly associated with progression-free and overall survival, respectively (Figure 1). For each gene in the Cox regression analysis, if the association was significant, the tumors were classified into low or high gene expression group using the median gene value as the cutoff. Survival curves were generated for the low or high gene expression tumors using the Kaplan-Meier method and log-rank test was used to evaluate if the survival curves were statistically different.

Patient characteristics and risk factors were compared using Fisher's exact test, Pearson's chi-squared test and Wilcoxon rank sum, when appropriate. Progression-free survival was compared between specimens with differing type I collagen IHC staining patterns also using log-rank test and Kaplan-Meier method. Analysis of SHG maximum signal and fiber CR were performed using Wilcoxon rank sum test. All tests were performed with two-sided tests and statistical significance was defined as p < 0.05, unless otherwise specified.

## SUPPLEMENTARY TABLE


