# Supplementary material for: Positive association of collagen type I with non-muscle invasive bladder cancer progression
Source: Oncotarget. 2016 Sep 17;7(50):82609–19. doi: 10.18632/oncotarget.12089 (PMC5347718; doi:10.18632/oncotarget.12089)
Supplement: Supplementary file 1 [file oncotarget-07-82609-s001.pdf]

Positive association of collagen type I with non muscle-invasive bladder cancer progression

SUPPLEMENTARY TABLE

Supplementary Table S1: Table summarizing the hazard ratio and *P*-value of the individual probes for *COL1A1* and *COL1A2* genes when analyzed by COX regression in correlation with overall survival or progression-free survival

	Overall survival		Progression-free survival	
**Probe**	**Hazard Ratio**	***P*-value**	**Hazard Ratio**	***P*-value**
COL1A1_8	2.175716363	*0.028048935*	3.043310304	*0.007992752*
COL1A1_9	1.930753099	*0.036279064*	2.235337961	*0.032386207*
COL1A1_32	3.509294636	*0.00377532*	2.742629329	*0.049807318*
COL1A1_56	2.343312142	*0.014400369*	3.943777057	*0.000679455*
COL1A1_57	1.779663758	0.065562	2.129381551	*0.045527236*
COL1A1_80	2.864238669	*0.009148189*	2.773971078	*0.030522618*
COL1A1_1746	1.892953437	0.101648105	1.321049901	0.57889439
COL1A1_1769	0.501702747	0.211642543	1.042121809	0.948933935
COL1A1_1770	1.372144138	*0.009512276*	1.47245198	*0.006983827*
COL1A1_1794	1.656796625	0.19635695	1.189960696	0.724799597
COL1A1_1817	0.402720759	0.117086068	0.817582121	0.769107749
COL1A1_1818	1.378448216	*0.005748492*	1.491216387	*0.003197269*
COL1A1_1846	1.618684533	*0.010406251*	1.671428827	*0.024333179*
COL1A1_1894	1.52734556	*0.019087522*	1.726584761	*0.011564214*
COL1A2_1066	1.298841865	*0.00634668*	1.248539726	0.070645498
COL1A2_1089	1.28815685	*0.002077654*	1.392265726	*0.000488009*
COL1A2_1090	1.199656803	0.102557911	1.31424364	*0.042214431*
COL1A2_1114	1.31414347	*0.003663674*	1.278422167	*0.033650109*
COL1A2_1137	1.329234268	*0.000657723*	1.432308353	*0.000314069*
COL1A2_1138	1.195670622	0.117933034	1.257763782	0.105938403

Red color in italic fonts indicates probes with a statistically significant P-value.
